# Myopia: Mechanisms and Strategies to Slow Down Its Progression

**DOI:** 10.1155/2022/1004977

**Published:** 2022-06-14

**Authors:** Andrea Russo, Alessandro Boldini, Davide Romano, Giuseppina Mazza, Stefano Bignotti, Francesco Morescalchi, Francesco Semeraro

**Affiliations:** ^1^Eye Clinic, Department of Neurological and Vision Sciences, University of Brescia, Brescia, Italy; ^2^Centro Oculistico Bresciano, Brescia, Italy

## Abstract

This topical review aimed to update and clarify the behavioral, pharmacological, surgical, and optical strategies that are currently available to prevent and reduce myopia progression. Myopia is the commonest ocular abnormality; reinstated interest is associated with high and increasing prevalence, especially but not, in the Asian population and progressive nature in children. The growing global prevalence seems to be associated with both genetic and environmental factors such as spending more time indoor and using digital devices, particularly during the coronavirus disease 2019 pandemic. Various options have been assessed to prevent or reduce myopia progression in children. In this review, we assess the effects of several types of measures, including spending more time outdoor, optical interventions such as the bifocal/progressive spectacle lenses, soft bifocal/multifocal/extended depth of focus/orthokeratology contact lenses, refractive surgery, and pharmacological treatments. All these options for controlling myopia progression in children have various degrees of efficacy. Atropine, orthokeratology/peripheral defocus contact and spectacle lenses, bifocal or progressive addition spectacles, and increased outdoor activities have been associated with the highest, moderate, and lower efficacies, respectively.

## 1. Introduction

Myopia is the most widespread refractive error and is principally due to the increasing axial length of the eyeball. In myopia, the distant object's image is formed anterior to the retinal plane, leading to blurred vision, which requires correction for clear vision. Noncorrected myopia impairs the patients' quality of life, affects school performance, and limits employability. Even corrected myopia may be responsible for serious complications such as staphyloma (outpouching of the back wall of the eye), glaucoma, cataract, choroidal neovascularization, retinal tears, schisis, and detachment; these complications together account for great economic implications for public health. Hence, many researchers and ophthalmologists have focused on myopia development and treatment.

A global increase in myopia cases has garnered renewed interest. In 2000, myopia affected 1.4 billion people worldwide, while in 2050, the number is estimated to reach 4.8 billion [1]. Myopia cases are increasing in Asian and Western countries. Higher prevalence has been reported among schoolchildren in East Asia, Singapore, China, Taiwan, and South Korea [2, 3]. A recent meta-analysis including 61,946 adults showed that in Europe, myopia increased from 17.8% (95% confidence interval (CI): 17.6–18.1) to 23.5% (95% CI: 23.2–23.7) in people born between 1910 and 1939 in comparison to those born between 1940 and 1979 (*P* = 0.03) [4]. A significant difference in the myopia incidences based on sex was found in most studies; however, the Correction of Myopia Evaluation Trial (COMET) study suggested that males showed slower progression [5]. Further, among females, myopia progressed differently at menarche. A study by Xu et al. in China reported a 13% higher risk of myopia in premenarche girls when adjusted for the exact age and behavioral risk factors [6].

Many etiological studies have assessed the role of both genetic and environmental factors in the development of myopia. Studies have reported a greater risk of myopia development in children with myopic parents. The Northern Ireland Childhood Errors of Refraction (NICER) study showed that the risk of myopia recurrence was 2.91 and 7.79 times more in children with one and two myopic parents, respectively [7]. Another study reported a 7.6, 14.9, and 43.6% myopia risk in children with none, one, and two myopic parents, respectively [8].

Myopia can be classified as syndromic and nonsyndromic. A known genetic factor has been implicated in genesis and development of syndromic myopia (such as Marfan syndrome or congenital stationary night blindness). Nonsyndromic myopia has no clear association with a genetic mutation; however, polymorphisms in different genes are associated with nonsyndromic myopia. A recent genome-wide association study named CREAM found 24 loci associated with myopia, which increase the myopia risk up to 10 folds.

Many studies have suggested that the environment plays a pivotal role in the development of nonsyndromic myopia forms; associations have been found with time spent in outdoor activities or near work, use of LED lamps for homework, population density, socioeconomic status, and use of video terminals. To control the deterioration of visual acuity, studies in recent decades tested several methods such as the use of anticholinergic drugs, correction of refractive error, multifocal spectacles or contact lenses, orthokeratology, and refractive surgery.

The growing interest in understanding myopia is justified due to possibility of stopping or slowing the disease through concrete mitigation strategies or new therapies. This review provides a critical analysis of the association between myopia development and environmental factors and analyzes the available strategies to reduce myopia evolution in children.

## 2. Outdoor Time and Near Work

Many studies have focused on the relationship between myopia development and progression and environmental factors such as near work, outdoor activities, sports practice, and use of technological devices. Most of these studies have suggested its inverse relationship with outdoor activities/sports and direct relationship with near work. Eppenberg and Sturm aiming to assess the protective role of outdoor light exposure in the incidence and prevalence of myopia recently summarized data from two cross-sectional studies, seven prospective cohort studies, and three intervention studies published between October 2008 and January 2019. The articles represent data of 32,381 participants between 6 and 18 years of age. Five of the nine cross-sectional studies found an inverse association [9]. Further, studies by Dirani and Sun revealed a significantly lower incidence of myopia in patients who reported a longer outdoor time (the reported odds ratio (OR), 0.90 (95% CI: 0.84–0.96, *P* = .004) and 0.74 (95% CI: 0.53–0.92, *P* < 0.001), respectively). Dirani et al. also reported that the mean amount of time of playing outdoor sports resulted to be longer among subjects without myopia (0.85 h/day, SD 0.80) than among those with myopia (0.72 h/day, SD = 0.82) (*P* = 0.007). Outdoor activities were associated with a lower prevalence of myopia; conversely, indoor sports were not. The data support the role of the overall outdoor activity as compared to sports alone in reducing the incidence of myopia [10, 11].

Jones-Jordan et al. examined 514 children and found that nonmyopic children were engaged in a significantly greater amount of sports and outdoor activities than the myopic ones (11.65 (SD 6.97) vs 7.98 (SD 6.54)) hours per week (*P* < 0.001) [12].

Conversely, a cohort study by Jacobsen et al. suggested that physical activity per sec is inversely associated with a refractive change toward myopia (*P* = 0.015) [13].

A systematic review assessing the correlation of physical activity, comprising the data from 263 studies, identified a solid relationship of more physical activity and lower myopia, but no evidence of physical activity as an independent risk factor for myopia was obtained. Hence, as per evidence, outdoor time remains the most important factor [14].

Chen et al. reported a later onset of myopia in people who spent more time outside. Guggenheim and Saxena confirmed this data (the relative risk reported was OR = 0.90 (95% CI: 0.45–0.96) and *R* = 0.54 (95% CI: 0.37–0.79; *P* = 0.002)) [15, 16]. Wu et al. showed a slower myopic shift in children who were encouraged to spend more time outside. (OR 0.46 (95% CI: 0.28–0.77); *P* = 0.003) [17]. However, studies by Jordan-Jones et al. Ma et al., and Hsu et al. [12, 18, 19] reported no association between myopia and time spent outdoors.

A recent school-based, prospective, cluster-randomized trial was conducted to assess the relationship between time spent outdoors and the myopia onset/progression. A total of 6,295 children were randomized into a control group (*n* = 2,037), test group I (*n* = 2,329, 40 minutes outdoor time/day), or test group II (*n* = 1,929, 80 minutes outdoor time/day). The study failed to demonstrate any significant association between the time spent outdoor and myopia development or progression [20]. Jones-Jordan et al. did not observe any retardation in myopia development in children who spent more time outdoors, as reported by He et al. [12, 20].

Many studies have identified an inverse association between myopia development and progression and outdoor exposure; however, contrasting evidence has also emerged. This may be due to biases. First, the data on near work, outdoor activities, and related parameters in almost all published studies were obtained from questionnaires and lacked uniformity. Moreover, the results of the questionnaires were influenced by geography, culture, cognitive ability, and memory bias. The refraction data might have been influenced by measurement bias. Complete cycloplegic refraction was obtained in only a part of the studies by using different drugs (tropicamide vs. cyclopentolate); therefore, these refraction results could not be considered reliable for statistical analyses.

Nevertheless, existing evidence supports this association. The mechanism through which outdoor exposure may be responsible for lowering the incidence of myopia is explained by different hypotheses. Sunlight peaks at a wavelength of 550 nm, resulting roughly to the peak of sensitivity of the human eye. Indoor light peaks at a longer wavelength. Thus, most of the light beams received by the eye are focused behind the retina plane and might cause a situation similar to that of a negative lens. This phenomenon has proven to stimulate global growth in myopia [21].

Another hypothesis focused on the importance of dopamine release stimulated by sunlight. Animal models (one-day-old white Australorp cockerels) were used to verify the effect of a translucent diffuser placed over the eye and kept on a 12 : 12 light/dark cycle. These birds exhibited excessive axial length causing myopia; however, if the diffuser was removed for 3 hours during the light period, the axial length did not grow. In birds wearing a diffuser, intravitreal injection of dopamine blocked axial growth. Dopamine antagonists exerted the opposite effects [22, 23].

Myopia development and progression have been associated with higher educational levels and near work. The latter is considered a group of activities performed at short working distances such as reading, studying, computer use, playing videogames, or watching TV. School children spend a lot of time in near vision activities, and this could be regarded as a risk factor for myopia development. To study the effect of near work, a meta-analysis was conducted comprising the available literature published between April 1, 1989, and May 1, 2014, with a total of 10,384 participants aged 6–18 years. Results showed a pooled OR of 1.14 (95% CI: 1.08–1.20), advocating that near activities are associated with myopia. A subgroup analysis based on the definition of near work found that children who performed more near work were more likely to be myopic (OR = 1.85; 95% CI: 1.3–2.62; *I*^2^ 85%) and that the odds ratio of myopia increased by 2% (OR = 1.02; 95% CI: 1.01–1.03; *I*^2^ 42.8%) for every diopter-hour increase of near work per week [24].

**Figure 1:**
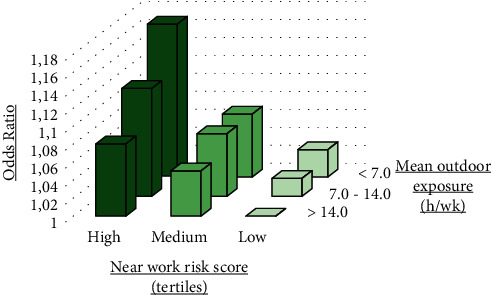
Odds ratios for near activity risk and the mean outdoor time on myopia at the age of 9 years. Near activities risk tertiles represent the combined risk of the computer use, reading, and reading distance. The outdoor time was classified into <7, 7–14, and >14 hours per week. The subset with low near risk and >14 hours per week of outdoor exposure was the reference subset (adapted from the study by Enthoven et al.).

The Generation R Study conducted in Rotterdam tested the relationship between computer use and myopia development. This study comprised a total of 5074 children born in Rotterdam between 2002 and 2006. Data on computer use and outdoor exposure were acquired at the age of three, six, and nine years using a questionnaire; reading time and reading distance were assessed at nine years of age. Statistical analysis showed a significant association between computer use at the age of 3 years and myopia at six and nine years (OR = 1.005, 95% CI: 1.002–1.010; OR = 1.009, 95% CI: 1.002–1.0017). The cumulative time of the computer use in infancy was significantly correlated with myopia at nine years (OR = 1.005, 95% CI: 1.001–1.009). In the same study, reading time at the age of nine years was significantly associated with myopia at nine years and axial elongation. The study found that the effect of near vision activities decreases longer outdoor exposure (Figure 1) [25].

A prospective study by Oner et al. found that only reading and writing had a negative association with annual myopic progression (*r* = −0.362, *P* = 0.010), while computer use, watching television, and outdoor activities had no correlation with the annual myopia evolution rate. Different near vision activities could differently affect myopia risk at different light levels, word sizes, and working distances [26].

According to Pӓrssinen and Lyyra, a correlation was found between time spent on reading or near work and myopia [27]. Conversely, the studies of Tan et al. reported no statistically significant associations between myopia progression and near activities in children [28, 29]. Contrasting evidences could be due to the difference in the age of the participants in the groups analyzed.

While accommodation and convergence occurring after prolonged near work have been proposed as the mechanisms for the development of myopia, a strong association between accommodation and myopia has not been found [27]. Forced hyperopic defocus has been shown as a significant stimulus for eye growth in experimental studies [30].

The coronavirus pandemic (COVID-19), a problem affecting people worldwide since the beginning of 2020, has changed people's habits and led to an increase in use of digital devices owing to lockdown measures. In order to establish the risk of increase in the incidence of myopia with the increased digital device use, Wong et al. reviewed studies published on the association between PC, tablet, or smart phone use and myopia. They found that current evidence is inconclusive, but most of the pieces of evidence suggest a higher risk of myopia in people spending more time on digital screens. They argued that the COVID-19 pandemic outbreak period could potentially aggravate myopia by increasing exposure to digital devices. Moreover, the usage of digital devices might have a long-term negative impact [31].

To limit the consequences, the American Ministry of Education recommends spending less than 20 minutes per day on electronic homework and prohibition of phones and tablets in classrooms [32].

Interestingly, the exposition to the red light (650 nm wavelength) at home with a desktop light therapy device had recently been shown to be effective in myopia control. At the 12-month follow-up visit, the group given red light therapy had a 70% reduction in myopia progression and 32% of patients in this group also had a ≥0.05 mm *axial length shortening* [33]. Further studies with double-masking and the placebo-controlled groups are needed to understand the long-term efficacy and safety, possible rebound effects, and optimal treatment strategies, beyond the potential underlying mechanisms.

## 3. Pharmacological Strategies

### 3.1. Atropine

Atropine, a nonselective muscarinic antagonist drug, is known for its potential myopia-inhibiting capacity. Initially, since accommodation was considered an important factor in myopia progression, atropine was used because of its cycloplegic effect. However, animal studies have revealed that the effect of atropine might be mediated by nonaccommodative mechanisms [34, 35].

Atropine has affinity for all five subtypes of acetylcholine receptors (MR1-MR5), which are distributed in different ocular tissues and scleral fibroblasts [36]. Several studies have shown that mAChR antagonists inhibit scleral proliferation in mice and humans and subsequently inhibit axial elongation of the eye [37].

Nonetheless, the exact mechanism by which atropine exerts its suppressive action on myopia has not been established yet. Some studies have demonstrated an increase in retinal dopamine after instillation of atropine and postulated that dopamine may stimulate the release of nitric oxide as a part of the signaling chain [38]. Recently, Barathi et al. suggested that GABAergic-mediated signaling is involved, while Carr et al. described a possible implication of *α*2 adrenergic receptors [39, 40].

Prepas proposed that pupil dilatation induced by antimuscarinic drugs leads to increased UV exposure, which controls the scleral growth through collagen cross-linking [41]. However, this hypothesis disagrees with the lack of myopic progression control after instillation of tropicamide [42].

Several randomized clinical trials have shown that 1% and 0.5% atropine are effective in slowing myopia progression [42–45]. The Atropine in the Treatment of Myopia (ATOM) study was a randomized, double-masked, placebo-controlled trial conducted in Singapore with over 400 children aged 6 to 12 years. For two years, 1% atropine eye drops were instilled, followed by a one-year suspension. The results after two years demonstrated a 77% reduction in progression of myopia as compared to the control group (−0.28 ± 0.92 diopters (D) compared with −1.20 ± 0.69 D in the placebo group with *P* < 0.001), but no change in the axial length compared to the baseline (−0.02 ± 0.35 mm) [43].

During the washout phase, the suspension of treatment caused a rebound effect in both refraction and axial length in the eyes treated with atropine, but the final progression was lower in the atropine-treated group than that of the control group [46]. Moreover, 1% atropine caused side effects such as photophobia, blurred vision, and reduced accommodation. However, the safety profile of a high dosage of atropine is a major concern in clinical practice, and reduced accommodation may require children to wear bifocal or progressive lenses to read. Recent clinical trials have confirmed that atropine is effective in controlling myopic progression with a dose-related effect.

In a two-year study conducted by Shih et al., 200 Taiwanese children were treated with 0.5%, 0.25%, or 0.1% atropine. After two years, there was a reduction in myopia progression by 61%, 49%, and 42% respectively, as compared with children treated with tropicamide in the control group (−0.04 ± 0.63 D/Y, 0.45 ± 0.55 D/Y, and 0.47 ± 0.91 D/Y in the 0.5, 0.25, and 0.1% atropine groups, respectively, in comparison to the control group (−1.06 ± 0.61 D)) [42].

The ATOM 2 study evaluated the efficacy and side effects of lower doses of atropine on myopic progression (0.5%, 0.1%, and 0.01% atropine instilled for 24 months followed by the 12-month washout phase). The authors demonstrated a dose-related effect, with higher doses leading to greater inhibition of myopia progression (−0.30 ± 0.60 D, −0.38 ± 0.60 D, and −0.49 ± 0.63 D in the 0.5%, 0.1%, and 0.01% atropine groups, respectively, (*P* = 0.02, between the 0.01 and 0.5% groups; *P* = 0.05, between other concentrations)) [47].

However, after suspension of treatment, there was a greater rebound effect in the eyes treated with higher concentrations of atropine, whereas only a slight increase was observed in the 0.01% group. After 36 months, myopia progression in the 0.01% group was −0.72 ± 0.72 D, while in the 0.5% and 0.1% groups it was −1.15 ± 0.81 D and −1.04 ± 0.83 D, respectively, (*P* < 0.001) [48]. The authors concluded that the lowest (0.01%) concentration seems to be the safest choice causing fewer adverse effects compared to higher formulations while retaining similar efficacy [47].

In a recent study of low-concentration atropine for myopia control (LAMP), Yam et al. compared 0.05%, 0.025%, and 0.01% atropine eye drops and described a dose-related effect on myopia progression. Atropine (0.05%) was the most effective in limiting both the spherical equivalent and axial elongation progression [49]. After two years, efficacy of 0.05% doubled than that of 0.01% atropine [50]. Regarding combined treatment with both atropine and multifocal or bifocal lenses, studies found a lower rate of myopic progression with both 1% and 0.5% atropine plus multifocal and bifocal lenses compared to placebo plus single-vision lenses [42, 50]. The most recent report from the same study (LAMP, Phase 3) regarding the third year of usage confirmed that atropine treatment achieved a better effect across all concentrations compared with the washout regimen. In particular, 0.05% atropine remained the optimal concentration over 3 years in the study population. The differences in rebound effects were clinically small across all 3 studied atropine concentrations. Stopping treatment at an older age and lower concentration is associated with a smaller rebound: the older the subject's age, the smaller the rebound effect. This might be explained by the slower inherent physiological progression of children at older ages, as previously demonstrated by the results of the LAMP study Phases 1 and 2 [51].

In conclusion, results from studies have proved that atropine eye drops, alone or in combination with other treatments, are useful in reducing myopic progression, although mild side effects were described, including pupil dilation, photophobia, and near blur. To date, atropine treatment has been adopted in Asian countries, such as Taiwan and Singapore.

### 3.2. Pirenzepine

Several studies have demonstrated that pirenzepine, a selective M1 muscarinic receptor antagonist, is effective in controlling the progression of myopia in children [52–54]. A study conducted on myopic Asian children treated with a pirenzepine 2% gel twice daily found a 44% reduction in myopic progression compared with the control group.

A parallel-group, placebo-controlled, double-masked, randomized trial conducted by Siatkowski et al. found a 41% reduction in myopic progression in children treated with a 2% pirenzepine gel compared with the placebo (0.58 D vs. 0.99 D after two years), but the difference in the axial length between the study groups was statistically insignificant. The United States-based clinical trial found that pirenzepine was well tolerated with mild to moderate adverse effects [53]. However, pirenzepine is not available as a treatment option currently.

### 3.3. 7-Methylxanthine

7-Methylxanthine, a nonselective adenosine antagonist, has been adopted as a treatment option only in Denmark. Oral administration of 7-methylxanthine causes a rise in the scleral collagen fibril diameter, amino acid content, and thickening of the sclera in rabbits [55].

A trial evaluated the effect of 400 mg 7-methylxanthine once a day in children compared to a placebo group. The results revealed a modest effect on myopia progression in children with moderate axial growth rates at the baseline (22%), but no effect in individuals with high-progressing myopia. The treatment seemed safe, with no ocular or systemic side effects [56]. Currently, 7-methylxanthine is a nonregistered drug in Denmark. Evaluation conducted on animals [57, 58] and humans have exhibited potential efficacy; however, further evaluations are needed.

## 4. Surgical Strategies

Refractive surgery was first used in a pediatric population in the 90s [59], with the aim to improve vision in a selected group of visually impaired children [60]. In the adult population, refractive surgery is used to achieve the best-uncorrected vision possible.

Amblyopia is a reduction in visual acuity or visual deprivation without an organic cause due to abnormal interaction between the two eyes and the brain. In a population-based cross-sectional study [61], amblyopia accounted for 33% of monocular visual impairment in children.

The most frequent cause of amblyopia is anisometropia. Myopic anisometropia of more than 2 D results in an increased incidence of amblyopia and reduced stereopsis. Anisometropia greater than 6 D is amblyogenic in all children [62]. Moreover, a higher degree of anisometropia affects amblyopia therapy and leads to a worse visual outcome [63].

Glasses, contact lenses, and patching are the most common options for treating pediatric high refractive errors associated with amblyopia. However, children may refuse conventional therapy for different reasons. If a significant refractive difference exists between the two eyes, the use of a spectacle may result in aniseikonia and interfere with good stereopsis. Correction with glasses, especially those with high refractive errors, may lead to a narrower field of view, prismatically induced aberrations, and social stigma. Contact lenses offer a better quality of vision and a larger field of view but are associated with poor compliance due to intolerance and difficulty of insertion and removal [64].

In a study by Paysse, factors associated with failure of traditional therapy are age >6 years, poor compliance, inadequate parental understanding, initial visual acuity of 20/200 or lower, and presence of astigmatism >1.5 D [65]. Children with craniofacial and/or ear abnormalities, hearing aids, or neurobehavioral disorders may be averse to wearing spectacles. These children can develop very poor vision in the amblyopic eyes because conventional treatment is more challenging [66].

Moreover, some studies have shown that only about two-thirds of cases with anisometropic amblyopia achieve good visual outcomes if treated with conventional methods [65, 67, 68]. If myopic anisometropia is more than 6 D, the chance of achieving a best-corrected visual acuity of 20/40 or better is only 25% [63].

The application of refractive surgery in the treatment of anisometropic amblyopia in children is still unclear. Options include laser vision correction such as photorefractive keratectomy (PRK), laser-assisted subepithelial keratectomy (LASEK), laser-assisted in situ keratomileusis (LASIK), or phakic intraocular lens implantations (anterior or posterior chamber). PRK, LASEK, and LASIK yield successful outcomes in refraction and visual acuity in children with high myopic anisometropia and amblyopia than in those who are noncompliant with traditional treatment [59, 69–82].

Nucci and Drack evaluated the safety and efficacy of refractive surgery in children with unilateral high myopia to supplement optical correction. A total of 14 eyes in 14 children aged 9–14 years received surgery (11 PRK and three LASIK). The preoperative best-corrected visual acuity was 20/147, while that at 20 months was 20/121. Average preoperative and postoperative refraction (spherical equivalent) was −7.96 ± 2.16 D and −0.67 ± 0.68 D at 20 months, respectively. Only minimal corneal haze was reported [73].

Autarata and Rehurek evaluated the results of PRK for high myopic anisometropia and contact lenses intolerance in 21 children aged 7–15 years. The mean preoperative and postoperative refraction was −8.93 ± 1.39 D and −1.66 ± 0.68 D, respectively (*P* < 0.05). A total of nine eyes gained one line of the best-corrected visual acuity, and five eyes gained two lines. No significant complications were observed. The authors concluded that PRK is safe and effective over a four-year follow-up period [83].

Phillips et al. treated LASIK myopic anisometropia in five patients between 8 and 19 years of age and evaluated the results over 18 months. The mean preoperative refractive error was −9.05 D, while the mean postoperative refractive error was −1.17 D, and two of five patients gained one line of vision [84].

In an analysis of 17 case series published by Daoud et al., 298 patients were treated with PRK, LASEK, and LASIK for severe myopic anisometropia. Follow-up ranged from 12 to 36 months. Patients' preoperative refraction was between −14.9 and −6 D and age varied between 0.8 and 19 years. The authors found an improvement in the best-corrected visual acuity from 20/30 to 20/400 preoperatively to 20/26–20/126 postoperatively. Improved binocular vision after surgery was found in 64% of patients in six of the largest studies analyzed [64]. Interestingly, several studies reveal an increased level of stereopsis after excimer refractive surgery [80, 81, 85].

Paysse evaluated the long-term visual acuity and the refractive outcome in 11 children who underwent PRK for the treatment of anisometropic amblyopia. She reported a long-term reduction in the refractive error with increased visual acuity. Stereoacuity improved in 55% of testable children [80].

Astle et al. found an improvement in the best-corrected visual acuity in 63.6% of children treated with LASEK. Positive stereopsis was present in 39.4% of patients preoperatively and 87.9% postoperatively [81]. In a retrospective study, Magli et al. evaluated the use of PRK in the treatment of 18 myopic anisometropic children. Best-corrected visual acuity showed an improvement after surgery (from 20/70 to 20/50), and the level of stereopsis increased in two of 18 patients [85].

Excimer laser surgery has also been successfully used to treat high bilateral myopic amblyopia. In a case study published by Astle et al., 11 patients aged 1–17 years were treated with LASEK. The average spherical equivalent was −8 D preoperatively and −1.2 D postoperatively. The average best-corrected visual acuity was 20/80 preoperatively and 20/50 postoperatively [76]. Tychsen reported nine patients between 3 and 16 years of age were treated with LASEK. After surgery, uncorrected acuity improved in all eyes, with improvement in behavior and environmental visual interaction [86].

Corneal haze is the predominant complication of ablative refractive surgery. In a meta-analysis [87], LASIK patients had lower rates of postsurgical haze than those of PRK (5.3% vs. 8.5%, respectively). In children, postsurgical haze is more common than in adults, given that children have a stronger inflammatory response. Long-term corticosteroids and mitomycin C have been recommended to reduce the incidence of postsurgical haze [88].

Patient cooperation may be challenging in the case of children. During laser or intraocular refractive surgeries in the adult population, the patient is asked to fixate on the operating light or laser target. Collaboration varies in children as they may not be able to fixate, and general anesthesia might be required. However, adolescents are often able to fixate [84]. Some studies have investigated the use of different anesthesia protocols during excimer laser surgery [89, 90].

However, according to Brown [91], given that the patient's line of sight is determined by the desire to actively fixate on an object, an unconscious patient is not able to direct the fovea toward a target. Corneal refractive surgery should be centered on the intersection between the patient's line of sight and the cornea, while the laser firing axis is centered on the surgeon's line of sight. Tilting the laser firing axis relative to the patient's line of sight could result in optically asymmetric ablation. The best timing for performing refractive surgery is debatable, but studies suggest that the best results are shown when performed early [87].

However, eye modifications such as changes in the axial growth and lens thickness can affect long-term outcomes of early surgery. In laser refractive surgery, possible corneal biomechanical changes over time must be considered [92]. In young children, corneal strength has not been established, but there is evidence that the corneal strength increases with age [93].

Another concern is the myopic regression. Most of it occurs during the first year after surgery, with lesser regression over the following 2–3 years [80]. Daoud et al. observed a myopic regression of 1 D/year on average in children treated for myopic anisometropic amblyopia [64]. For these reasons, authors suggest overcorrecting and targeting slight hyperopia in myopic corrections [92].

Another option for surgery in children with high refractive errors and amblyopia is phakic intraocular lens implantation. The phakic intraocular lens was first used in the pediatric population in 1999 [94]. There are two types of FDA-approved phakic intraocular lenses: an anterior chamber phakic intraocular lens called Verisyse (Ophtec BV) in the United States, similar to the Artisan phakic intraocular lens in Europe and Asia and a posterior chamber phakic intraocular lens called Visian Implantable Collamer Lens (ICL) (Staar Surgical Co). The Visian ICL is implanted between the iris and the natural lens with the haptics located in the ciliary sulcus.

Indications of ICL implantation in the pediatric population are high anisometropia, myopia, or hyperopia noncompliant with conventional treatment, bilateral high ametropia noncompliant with conventional treatment, and high refractive amblyopia associated with neurobehavioral disorders [95, 96]. In recent years, several studies have been published on the use of anterior chamber phakic intraocular lenses for the treatment of refractive errors in children. These studies documented an improvement in uncorrected visual acuity, and surgery was well tolerated [97–99].

In a study conducted by Pirouzian et al., six pediatric patients with anisometropic myopic amblyopia underwent Verisyse anterior chamber phakic intraocular lens implantation. Patients were aged 5–11 years, and none of the patients were compliant with glasses or contact lenses. Results showed the improved best-corrected visual acuity from less than 20/400 to a mean of 20/70 postoperatively, an increase in stereopsis, and minimal side effects [97].

One of the most important concerns was the potential long-term endothelial cell loss. For these reasons, guidelines approve phakic intraocular lenses only when the anterior chamber depth is more than 3.2 mm. In the studies of Pirouzian et al. and Ip et al., the endothelial cell loss rate after 3–5 years of follow-up was between 6.5% and 15.2% [99, 100]. However, as with visual acuity, the endothelial count is difficult to measure in all children, and the real cell loss cannot be accurately assessed in these studies.

Since 2013, different authors have reported their experience with posterior chamber phakic intraocular lenses in children. Results showed an improvement in corrected and uncorrected visual acuity [101–103]. In 2017 large case series, Tychsen et al. published the results of Visian phakic intraocular lens implantation in 40 eyes of 23 children with high anisometropia and amblyopia. About 57% of the patients had a neurobehavioral disorder. Best-corrected visual acuity improved from 20/74 preoperatively to 20/33 postoperatively. Uncorrected visual acuity improved 25-fold, which is relevant, given that children with neurobehavioral disorders are intolerant to glasses. Moreover, 85% of the children had improved social performance [103].

Complications from the above-mentioned studies were due to the lens position, including a pupillary block from not enough patent peripheral iridotomy and pigment dispersion from the lens rubbing on the posterior iris [101–103].

There are several advantages of using phakic intraocular lenses compared to laser refractive surgery. The phakic intraocular lens procedure is reversible, and there is less risk of refraction regression over time. Moreover, laser surgery carries a risk of corneal haze. Nevertheless, there is a need for further studies on the long-term effects of phakic intraocular lenses on endothelial cells, the risk of cataract formation, and angle-closure glaucoma.

Despite evidence of efficacy and short-term safety, many questions about refractive surgery in children have not yet been answered. The major concerns to be explored are the lack of pediatric nomograms, the role of anesthesia, the lack of evidence regarding the effect of the eye growth on long-term outcomes, the instability of the refractive error in children, susceptibility to trauma, and lack of evidence of long-term safety.

## 5. Optical Strategies

Several strategies have been attempted in order to optically control the progression of myopia, including under and overcorrection. In China, two studies aimed to evaluate the progression of myopia in uncorrected eyes. In the first study proposed by Hu and Guo [104], 90 participants were divided into the three groups: uncorrected, monocular corrected, or binocular corrected. The results showed that over a 12-month follow-up visit, the uncorrected patients had a faster progression of myopia (−0.95 ± 0.12 D) as compared to those who were fully corrected (−0.50 ± 0.15 D). However, this study had some limitations: the selection procedure and age were not specified, and the groups were not well matched.

In another study, Sun et al. [105] evaluated a cohort of 121 twelve-year-old Chinese children. In the first year, in the uncorrected group, myopia progression was less (−0.39 ± 0.48 D) as compared to the full-corrected group (−0.57 ± 0.36 D; *P* = 0.03). This difference was significant even after adjusting for the baseline standard error of regression, age of the myopia onset, height, presence of parents with nearsightedness, and time spent in outdoor and indoor activities (−0.39 ± 0.06 D vs −0.58 ± 0.06 D, *P* < 0.01).

Lastly, Ong et al. [106] reported no difference in myopic progression over a three-year period among myopic children who wore full-corrected glasses full-time, part-time, or not at all.

### 5.1. Undercorrection of Myopia

Undercorrection is one of the optical strategies proposed to slow the progression of myopia. It is based on the rationale that in undercorrected eyes, the accommodative response for near vision is reduced [107]. In fact, in animal models (chicks, tree shrews, marmosets, and infant monkeys) [21, 108, 109], a myopic defocus, in which the retinal image is formed in front of the retina, was capable of inhibiting eyeball elongation and associated myopic progression.

Tokoro and Kabe [110] found that in a population aged 7–15 years, the rate of myopia progression was lower with undercorrection (−0.54 ± 0.39 D) than with full correction, either in full correction full-time wear (−0.75 D ± 0.27 D) or in full correction part-time wear (−0.62 ± 0.32 D). This study had several limitations, including a small sample size, limited statistical analysis, and concurrent use of pharmacological intervention for myopia control.

In the study by Li et al. [111], the study population consisted of 12-year-old Chinese children. One hundred-twenty patients were undercorrected, and 133 patients were fully corrected; at one year, no statistically significant difference was observed between the two groups. However, a regression analysis showed a significant association if the refractive error, not the axial length, was considered. In this case, the progression of myopia decreased with an increasing amount of undercorrection (*R*^2^ = 0.02; *P* = 0.02). However, in order to achieve reduction in myopia progression by 0.25 D, undercorrection of more than 1.50 D was required.

In both studies by Adler and Millodot [107] and Koomson et al. [112], undercorrection did not prove a statistically significant reduction in myopia progression. Adler and Millodot found that in a cohort of 48 children aged 6–15 years, undercorrection by 0.50 D was associated with myopia progression of 0.17 D when compared to full correction.

Koomson et al. enrolled 150 Ghanaian children who were divided into two groups (*n* = 75). The first group was undercorrected by 0.50 D, while the second group was fully corrected. At two years, myopia progressed by the same rate in both the groups (−0.54 D ± 0.26 in the full-corrected group vs −0.50 D ± 0.22 in the undercorrected group; *P* = 0.31). Conversely, three studies have reported that under-correction causes a more rapid progression of myopia.

Chung et al. [113] reported that 47 children undercorrected by 0.75 D had a greater progression of myopia compared with the 47 children who were fully corrected (−1.00 D vs 0.77 D; *P* < 0.01); however, the axial elongation was smaller in the undercorrected eyes (0.58 mm vs 0.65 mm; *P* = 0.04).

Chen [114] designed a study in which 77 fully corrected eyes were compared to 55 undercorrected eyes. The two groups were matched for the age, sex, and refractive error. At a 12-month interval, the undercorrected −0.25 to −0.50 D) group exhibited a significant myopic progression (−0.60 D vs −0.52 D; no standard deviation; standard error; and 95% confidence interval were reported).

Vasudevan et al. [115] retrospectively examined myopia progression rate records from the USA and the level of undercorrection of myopia versus full correction of myopia. They found that greater undercorrection was associated with a greater progression of myopia (*P* < 0.01).

In all these scenarios, both eyes were corrected, either undercorrected or fully corrected. However, two studies evaluated the rate of progression of myopia by correcting only one of the eyes.

In a population of 18 children aged 11 years, Phillips [116] noticed that undercorrection of the nondominant eye was associated with a slower progression of myopia compared to that in the dominant eye, which was fully corrected. The intereye difference was 0.36 D/y (*P* = 0.002).

However, Hu and Guo [104] reported opposite results, in which the undercorrection of one eye in myopic children was associated with a faster progression than fully corrected ones (−0.67 ± 0.22 D vs −0.50 ± 0.15 D).

Unfortunately, considering all human trials, the evidence supporting undercorrection as feasible for slowing the progression of myopia is low. Moreover, many pediatric practitioners suggest that the goal is to an attain optimal vision, which can be achieved by full correction.

### 5.2. Overcorrection of Myopia

In a case-control study by Goss [117], 36 children aged 7–15 years were overcorrected by 0.75 D and matched by control individuals randomly selected from the files of a university optometry clinic. The rate of progression among the groups was different but not statistically different; −0.49 D/year in the overcorrected group versus −0.47 D in the control group.

### 5.3. Bifocal and Multifocal Lenses

The rational use of bifocal or multifocal lenses to slow the progression of myopia is based on two theories. The first one, proven in animal models [108, 118], is based on central and peripheral hyperopic retinal defocus caused by a large accommodative lag [119, 120], which is defined as the residual refractive error of the difference between the accommodative demand required and its response. A large accommodative lag causes a hyperopic retinal defocus, which stimulates axial elongation in central defocus. Furthermore, in the case of peripheral defocus, the eye globe seems to acquire a more prolate shape. However, this stimulus is nullified by short periods of clear vision [21]; therefore, whether transient hyperopic retinal blur can lead to the onset and/or progression of myopia remains unclear.

The second theory assumes that during accommodation, there is a mechanical tension created by the crystalline lens or ciliary body. On the one hand, this tension restricts the equatorial ocular expansion, causing accelerated axial elongation; on the other hand, as the ciliary-choroidal tension increases, the effort needed to accommodate increases as well. This probably leads to a further increase in accommodative lags in children, which is a consequence rather than a cause of myopia [121–125]. Regarding the association between myopia in children and accommodative lags, it has been reported that

1. Compared to emmetropic children, myopic children generally show insufficient accommodation with larger accommodative lags, even before the development of myopia. [120, 123, 126, 127].
2. In myopic children, a larger accommodative lag correlates with a faster myopia progression [128]

Unfortunately, as seen in the undercorrection approach, no consensus exists regarding the use of bifocal or multifocal lenses to slow the progression of myopia. This is mainly due to the standard near addition power use in the trials, typically between +1.00 D and +2.00 D so that interindividual differences are nullified, causing even a possible overcorrection in some cases.

The COMET study was a randomized, multicenter clinical trial in which 469 children, aged 6–11 years, were enrolled and divided into two groups: the first group was assigned to progressive addition lenses (with +2.00 D addition) and the second group to single-vision lenses. At three years, the difference between the progressive addition lenses and the control group in diopters was 0.20 ± 0.08 D and the axial elongation was 0.11 ± 0.03 mm. Even if statistically significant, these differences were considered clinically insignificant [129].

The same conclusions were obtained in the COMET 2 study [130]. A total of 180 children aged 8–12 years with spherical equivalent refraction from −0.75 D to −2.50 D and near esophoria ≥2 prism-diopters were enrolled. An additional inclusion criterion was high accommodative lag, initially set to at least 0.50 D (accommodative response less than 2.50 D for a 3.00 D demand) and subsequently restricted further to at least 1.00 D. A total of 110 children completed the study in three years; the progression of myopia was −0.87 D in the group treated with progressive addition lenses (+2.00 D) versus −1.15 D in the single-vision lens group. Nevertheless, despite being statistically significant, the authors considered the results to be clinically insignificant.

Cheng et al. [131] attempted to evaluate the use of bifocal and prismatic bifocal lenses. One hundred thirty-five Chinese-Canadian children, aged 8–13 years with myopia progression of at least 0.50 D in the preceding year, were randomly assigned to one of the three treatments: single vision (control, *n* = 41), +1.50 D executive bifocals (*n* = 48), and +1.50 D executive bifocals with 3-Δ base-in the prism in the near segment of each lens. At the three-year follow-up, the progression of myopia in terms of diopters and axial length elongation was highest in children treated with single vision (−2.06 D and 0.82 mm) compared to those who were treated with bifocal (−1.25 D and 0.57 mm) or prismatic bifocal lenses (−1.01 D and 0.54 mm). Furthermore, in children with high accommodative lags (>1.00 D), no difference was observed in myopia control using bifocal or prismatic bifocal lenses. Instead, in children who showed low lags of accommodation (≤1.00 D), greater benefits were observed using prismatic bifocal lenses. According to the authors, this could be explained as prismatic bifocal lenses, because prisms may reduce the convergence and lens-induced exophoria with prism correction.

Currently, research is moving from the correction of the hyperopic shift to the induction of myopic peripheral defocus. The rationale is based on two findings.

**Figure 2:**
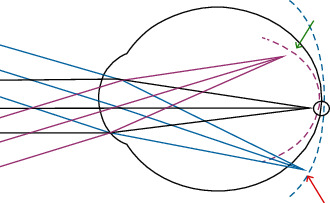
Peripheral hyperopic defocus (red arrow) might lead to axial elongation. A myopic defocus (green arrow) can be achieved with orthokeratology, contact lenses, laser refractive surgery, and spectacle lenses (defocus incorporated multiple segment lenses and Apollo progressive addition lenses).

1. Visual signals derived from the peripheral retina are stronger than those originating from the central retina [132, 133]
2. Optical defocus in the peripheral retina governs ocular growth: peripheral defocus stimulates axial elongation of the eye, while the opposite effect is demonstrated with peripheral myopic defocus (Figure 2) [134–140]

Spectacles of two types can induce peripheral myopic defocus: The defocus incorporated multiple segment lenses and Apollo progressive addition lenses (Apollo PALs, Apollo Eyewear, River Grove, IL, USA)and defocus incorporated multiple segment (DIMS) lenses [141] are custom-made plastic spectacle lenses. Each lens includes a central optical zone (9 mm in diameter) for correcting distance refractive errors and an annular multifocal zone with multiple segments (33 mm in diameter) with a relative positive power (+3.50 D). The diameter of each segment is 1.03 mm. Lam et al. [141] evaluated the use of defocus incorporated multiple segments versus single-vision lenses in 160 children. The results indicated that myopia progressed slower by 52% in the defocus incorporated multiple segment group than that in the single-vision group (−0.41 ± 0.06 D in the defocus incorporated multiple segment group and −0.85 ± 0.08 D in the single-vision group; mean difference −0.44 ± 0.09 D, *P* < 0.001). Moreover, the axial elongation was shorter in children in the defocus incorporated multiple segment group (0.21 ± 0.02 mm) by 62% than those in the single-vision group (0.55 ± 0.02 mm in the defocus incorporated multiple segment; mean difference 0.34 ± 0.04 mm, *P* < 0.001). These preliminary results were confirmed after a 3-year follow-up, showing that the myopia control effect was sustained in the third year in children who had used the DIMS spectacles in the previous 2 years and was also shown in the children switching from single vision to DIMS lenses [142]. Interestingly, in a study by Zhang et al., [143] baseline relative peripheral refraction (RPR) was assessed as a variable on the myopia control effects in myopic children wearing DIMS lenses. The authors concluded that DIMS lenses slowed down myopia progression, and myopia control was better for the children with baseline hyperopic RPR than the children with myopic RPR. This may partially explain why the efficacy of DIMS technology varies among myopic children and advocates the need for customized myopic defocus for patients to optimize myopia control effects. Indeed, similar results were found in animal studies, showing that a greater hyperopic defocus leads to more myopia progression while inducing myopic defocus retarded myopia progression [144]. Outcomes in infant monkeys and chicks advocated that spatial resolution at the anatomic level of the optical pathway could modulate overall eye growth [145]. Animal studies using contact lenses with embedded myopic defocus found that myopia progression could be slowed by 20% to 60% [146, 147].

The Apollo progressive addition lenses comprise an asymmetrical myopic defocus design with a 3 myopic defocus zone, including a +2.50 D full-positive power superior zone, an 80% full myopic defocus power nasal zone, and a 60% full myopic defocus power temporal zone. Currently, a prospective, multicenter, randomized controlled trial, promoted by Li, is ongoing to evaluate the possible efficacy of the defocus incorporated multiple segment and Apollo progressive addition lenses [148].

### 5.4. Contact Lenses and Orthokeratology in Myopia Control

As previously reported, a theory for eye elongation suggests that axial elongation is caused by peripheral retinal hyperopic defocus [105, 135, 149, 150].

This theory has led researchers to consider that reducing peripheral hyperopic defocus or inducing peripheral myopic defocus with bifocal, progressive, or multifocal lenses may help prevent myopic progression. In animal models, evidence suggests that the imposition of hyperopic or myopic defocus with negative or positive power lenses, respectively, can influence eye growth and lead to compensatory refractive changes: hyperopic defocus leads to longer and more myopic eyes and myopic defocus leads to shorter and more hyperopic eyes [151–156].

This supports the theory of slowing down axial elongation with optical treatments that correct distance vision while achieving simultaneous myopic defocus.

The reduction of peripheral retinal hyperopic defocus by contact lenses represents a new and interesting area of research that could be an effective intervention in myopia control. Effective contact lens options for myopia control include multifocal, extended depth of focus (EDOF), and orthokeratology contact lenses.

### 5.5. Single-Vision Rigid Gas-Permeable and Soft Contact Lenses

Single-vision lenses intend to correct the refractive error and are not prescribed for myopia control [149, 150]. Over several decades, there have been suggestions that gas-permeable contact lenses (not orthokeratology design) can slow myopia progression in children, but these studies have shown important limitations in their study design [157–160]. Nevertheless, well-conducted studies have recently demonstrated that gas-permeable contact lenses have no effect on the progression of myopia in children [160], even among children who use them regularly. These lenses temporarily flatten the corneal curvature without affecting axial elongation.

Although Atchison [161] has revealed that spherical contact lenses produce more peripheral myopic shift than spherically surfaced spectacle lenses, some prospective randomized studies did not find any differences in the myopia progression rate between soft contact lenses and spectacle wearers [162, 163]. However, other studies have tried to compare rigid with soft contact lenses. Katz et al. [160] found no difference in myopia progression or axial elongation over a period of two years between children wearing gas-permeable and soft single-vision contact lenses. Walline et al. [162] reported no difference in the amount of axial elongation between gas-permeable and soft single-vision contact lens wearers.

### 5.6. Soft Bifocal, Peripheral Gradient, and EDOF Contact Lenses

**Figure 3:**
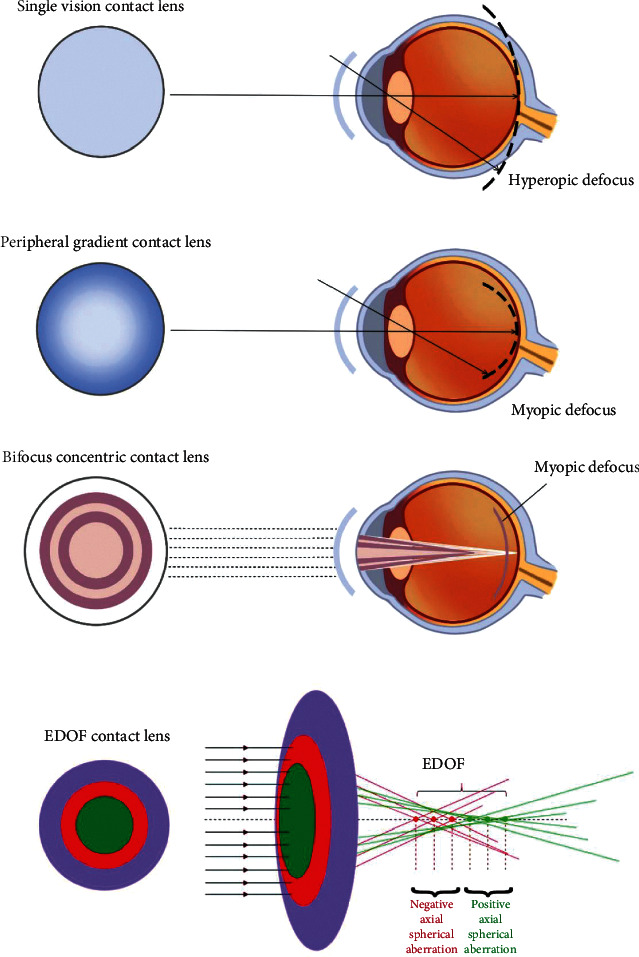
Single-vision contact lenses (CLs) provide a peripheral hyperopic defocus. A peripheral myopic defocus can be achieved with peripheral gradient CL, bifocal CL, and EDOF CL.

Three different promising types of contact lenses for myopia control in children have been studied: bifocal concentric lenses, peripheral gradient lenses, and EDOF contact lenses (Figure 3).

The first two multifocal contact lens designs include a central area for correcting myopia. However, bifocal concentric lenses use a concentric zone of rings with positive power addition to concurrently impose peripheral myopic defocus, and peripheral gradient lenses produce constant peripheral myopization defocus that increases gradually from the central optic axis toward the periphery [164]. The third type is based on the EDOF theory, which was designed to incorporate and manipulate selective higher-order aberrations (mainly spherical aberration) to achieve the global retinal image quality that was optimized for points at and anterior to the retina and degraded for points posterior to the retina. It was hypothesized that a poor image quality posterior to the retina prevents axial elongation [165].

Demonstrating the propensity for slowing both refractive and axial length myopia progression by around 30%–50% [166, 167], these contact lens options have the capability of correcting myopia as well as providing a treatment strategy for myopia control. In contrast, spectacle lens alternatives have shown less effective success for myopia control [168] except in one specific prismatic bifocal design [131] and a novel multisegment defocus design [141]. Moreover, in clinical studies, contact lenses provide better lens centration and are less affected by eye movements than spectacle lenses [135].

Data from two recent clinical pilot studies showed that adding myopic defocus to the distance correction reduced myopia progression by an average of 0.27 D/year after one year [147, 169], which is slightly better than the effect seen at one year using progressive addition lenses or bifocal lenses [129, 130, 170–172].

MiSight 1 day is a daily replacement of hydrophilic soft bifocal contact lenses approved by the FDA for correction of nearsightedness and slows its progression in children, aged 8 to 12 years, with a refraction of −0.75 to −4.00 D (spherical equivalent) and astigmatism less than or equal to 0.75 D at the beginning of treatment. MiSight's Activ Control™ technology is based on an optic zone concentric ring design. Concentric zones of the alternating distance and near power produce two focal planes, allowing for the correction of the refractive error and 2.00 D of simultaneous myopic retinal defocus. A two-year randomized clinical trial [164] showed lesser progression and axial elongation in the MiSight group than in the single-vision spectacle group.

Several studies [147, 164, 169, 173–178] published between 2011 and 2016 showed a reduction of 38.0% in myopia progression and 37.9% in axial elongation with multifocal soft contact lenses. In 2014, Benavente-Perez et al. [135] showed the effect of soft bifocal contact lenses on eye growth and the refractive state of 30 juvenile marmosets by imposing hyperopic and myopic defocus on their peripheral retina. Each marmoset wore one of three investigational annular bifocal contact lens designs in their right eye and a plano contact lens in the left eye as a control for 10 weeks. The three types of lenses had a plano center zone (1.5 mm or 3 mm) and +5 D or −5 D in the periphery (referred to as +5 D/1.5 mm, +5 D/3 mm, and −5 D/3 mm). The results were compared with untreated, single-vision positive and negative, and +5/−5 D multizone lens-reared marmosets. Eyes treated with positive power in the periphery showed to grow significantly less than untreated eyes and eyes with multizone contact lenses, supporting the use of bifocal contact lenses as an effective treatment for myopia control. Moreover, the treatment effect was associated with the size of the peripheral treatment zone as well as with the peripheral refractive state and the eye growth rate before the treatment started.

The bifocal lenses In nearsighted kids (BLINK) randomized clinical trial [179] has recently determined the role of soft multifocal lenses in slowing myopia progression in children, comparing high-add power (+2.50 D) with medium-add power (+1.50 D) and single-vision contact lenses. A total of 294 children with −0.75 D to −5.00 D of spherical component myopia and less than 1.00 D of astigmatism were enrolled, with a three-year follow-up. Adjusted three-year myopia progression was −0.60 D for high-add power, −0.89 D for medium-add power, and −1.05 D for single-vision contact lenses. This demonstrated that treatment with high-add power multifocal contact lenses significantly reduced the rate of eye elongation compared with medium-add power multifocal and single-vision contact lenses. However, further research is required to understand the clinical importance of these data.

EDOF contact lenses were tested in a three-year prospective, double-blind trial [165] that demonstrated their efficacy in slowing myopia progression. A total of 508 children with the cycloplegic spherical equivalent −0.75 to −3.50 were enrolled and randomized in one of the five groups: one group with single vision, two groups with bifocal, and two groups with EDOF contact lenses (configured to offer EDOF of up to +1.75 D and +1.25 D). At two years, the two groups of EDOF lenses slowed myopia by 32% and 26% and reduced axial length elongation by 25% and 27%, respectively. However, efficacy was not significantly different between the bifocal and EDOF lens groups.

### 5.7. Orthokeratology (Ortho-K) Lenses

Orthokeratology (ortho-k) is defined as a “reduction, modification, or elimination of a refractive error by programmed application of contact lenses [180].” It refers to the application of a rigid contact lens at night to induce temporary changes in the corneal epithelium shape, allowing for clear, unaided daytime vision. Wesley and Jessen in the 1950s casually observed spectacle blur experienced by patients after wearing hard contact lenses. This blurring was subsequently related to lens-induced epithelial reshaping, which was then utilized for therapeutic purposes [181].

**Figure 4:**
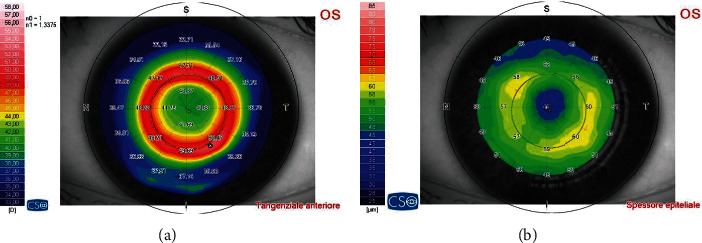
Epithelium remodeling is achieved with orthokeratology. Central corneal flattening is accompanied by a midperipheral steepening (tangential map, (a)), due to accumulation of the epithelium (epithelial thickness map, (b)).

Studies have shown that myopic orthokeratology lenses produce a flattening of the central cornea and a steepening of the midperipheral cornea, accompanied by changes in the epithelial thickness (Figure 4) [182–184].

Although these lenses were designed for refractive error correction, studies have revealed a secondary advantage of slowing myopic progression [149] by creating peripheral myopic defocus secondary to epithelial reshaping. A number of studies have shown a 30 %–71% reduction in axial elongation compared with the control [150, 185, 186].

Other studies and meta-analyses have revealed a 40%–60% mean reduction in the rate of refractive change compared with controls using spectacles to correct myopia [168, 187–194]. In one of the first trials, the retardation of myopia in orthokeratology study [195], axial elongation was reported to be slowed by an average of 43%.

In a second trial, the high myopia-partial reduction orthokeratology study [196], highly myopic individuals were enrolled and randomly assigned into partial reduction orthokeratology and single-vision spectacle groups. The first group needed to wear single-vision spectacles to correct residual refractive errors during the day. In this group, the axial elongation was 63% less than that of the second group. More recently, orthokeratology and gas-permeable lenses have been compared with a novel experimental study design [197]. Patients were fitted with overnight orthokeratology in one eye and traditional rigid gas-permeable lenses for daytime wear in the contralateral eye. The lenses were worn for six months. After a washout period of 2 weeks, lens-eye combinations were reversed and wearing lens was continued further for six months. The results revealed no increases in axial elongation over either the first or second six-month period for eyes with orthokeratology, compared with an increase in 0.04 mm and 0.09 mm, respectively, in eyes with gas-permeable lenses.

A recent one-year retrospective study by Na and Yoo [198] investigated myopic progression in children with myopic anisometropia who underwent orthokeratology treatment in their myopic eye and no correction in their emmetropic eye. The results showed statistically significant reduction in axial length elongation in the treated eye (0.07 ± 0.21 mm, *P* = 0.038) as compared with the control eye (0.36 ± 0.23 mm, *P* < 0.001).

Zhang and Chen [199] in a retrospective study compared the effect of toric versus spherical design orthokeratology lenses on myopia progression in children with moderate-to-high astigmatism (cylinder >1.5 D). Toric orthokeratology wearers had a 55.6% slower rate of axial elongation than that of the spherical group. Some studies have tried to assess the effects of combined treatments, such as orthokeratology lenses and atropine. Studies by Wan et al. [200] and Kinoshita et al. [201] found improvement in myopia control by combining the two strategies compared with orthokeratology monotherapy.

Although orthokeratology has a significant effect on slowing axial elongation, the results vary among individuals. Some patients show little or no myopic progression, while others continue to progress. Some studies [202–207] have shown that better myopia control is positively associated with a higher degree of baseline myopia, older age of the myopia onset and at initiation of treatment, larger pupil size, and a smaller resulting central optical zone (more peripheral myopia induced by a ring of steepening outside the treatment zone).

Cheung et al. [186] suggest that ideal candidates for orthokeratology might be children around 6–9 years of age with fast myopic progression (increase in the axial length of ≥0.20 mm/7 months or spherical equivalent of ≥1 diopter/year). Moreover, several studies have shown that children are sufficiently mature to safely and successfully wear different types of contact lenses, such as soft [208, 209] and orthokeratology lenses [191, 192].

## 6. Conclusions

The rapid increase in the prevalence of myopia, especially in Asian and Western countries, has made it a significant public health concern. In fact, high myopia (≥5 D or axial length ≥26 mm) is associated with an increased risk of vision-threatening complications such as retinal detachment, choroidal neovascularization, primary open-angle glaucoma, and early-onset cataract. Many studies have suggested the implication of both genetic and environmental factors in the development of myopia. The genetic pool is associated with both syndromic and nonsyndromic forms of myopia, whereas the environment plays an important role in nonsyndromic forms. However, we are far from understanding complex pathogenesis.

Various options have been assessed to prevent or slow myopia progression in children.

Environmental modifications, such as spending more time outdoors, can decrease the risk of the onset of myopia. In fact, many studies have identified an inverse association between the myopia onset and progression in outdoor exposure and a direct association with near work. However, contrasting evidence has also emerged, perhaps because of many biases, such as recall and measurement bias.

Optical interventions such as bifocal/progressive spectacle lenses, soft bifocal/multifocal/EDOF contact lenses, and orthokeratology lenses show moderate reduction in the myopia progression rate compared to single-vision lenses. All of these options seem to reduce hyperopic peripheral defocus, which is a stimulus for axial elongation, thus promoting myopic peripheral defocus and slowing axial elongation.

Regarding spectacle lenses, promising results are derived from the use of defocus incorporated multiple segment lenses and progressive addition lenses. However, further studies are needed to confirm this hypothesis. Conversely, undercorrection of the myopic refractive error does not slow the progression of nearsightedness. In fact, several studies have revealed no difference in progression with undercorrection. Others have reported an increase in myopia progression compared with full correction; thus, the full correction of myopia is currently recommended to attain an optimal vision as the main aim.

Gas-permeable and soft single-vision contact lenses are prescribed solely to correct the refractive error because many studies have shown no effects on axial elongation and myopia control.

Refractive surgery may be an interesting option for treating amblyogenic anisometropia in children who refuse conventional therapy. Despite its successful outcomes in refraction and visual acuity, the use of refractive surgery in these individuals remains unclear, mainly because of the need for anesthesia, susceptibility to trauma, lack of pediatric nomograms, instability of the refractive error, and lack of evidence of long-term safety. Further studies are needed to better explore the role of refractive surgery in this area.

Currently, pharmacological treatment with atropine is the most researched and effective strategy for myopia control. In particular, low-concentration atropine (0.01%) is known to maintain its efficacy on myopia control with a lower rate of side effects. Interestingly, data from studies on the effects of combined treatments, such as low-concentration atropine (0.01%) plus orthokeratology lenses or low-concentration atropine plus soft bifocal contact lenses (bifocal and atropine in myopia, BAM study), suggest that the combination seems to be superior to monotherapy. However, the BAM study is still ongoing, and no results have yet been published.

In summary, all these options for controlling myopia progression in children exhibit varying degrees of efficacy, as shown in the literature. Compared with single-vision spectacles as control, atropine exhibits the highest efficacy; orthokeratology, peripheral defocus contact, and spectacle lenses have moderate efficacy, whereas bifocal or progressive addition spectacles and increased outdoor activities show lower efficacy [185].

## Data Availability

No data were used to support this study.

## References

[1] Holden B. A., Fricke T. R., Wilson D. A. (2016). Global prevalence of myopia and high myopia and temporal trends from 2000 through 2050. *Ophthalmology*.

[2] Xiang F., He M., Zeng Y., Mai J., Rose K. A., Morgan I. G. (2013). Increases in the prevalence of reduced visual acuity and myopia in Chinese children in Guangzhou over the past 20 years. *Eye*.

[3] Ding B. Y., Shih Y. F., Lin L. L. K., Hsiao C. K., Wang I. J. (2017). Myopia among schoolchildren in East Asia and Singapore. *Survey of Ophthalmology*.

[4] Williams K. M., Bertelsen G., Cumberland P. (2015). Increasing prevalence of myopia in Europe and the impact of education. *Ophthalmology*.

[5] COMET Group (2013). Myopia stabilization and associated factors among participants in the correction of myopia evaluation trial (COMET). *Investigative Ophthalmology & Visual Science*.

[6] Xu R., Jan C., Song Y. (2020). The association between menarche and myopia and its interaction with related risk behaviors among Chinese school-aged girls: a nationwide cross-sectional study. *Journal of Developmental Origins of Health and Disease*.

[7] O’Donoghue L., Kapetanankis V. V., McClelland J. F. (2015). Risk factors for childhood myopia: findings from the NICER study. *Investigative Ophthalmology & Visual Science*.

[8] Ip J. M., Huynh S. C., Robaei D. (2007). Ethnic differences in the impact of parental myopia: findings from a population-based study of 12-year-old Australian children. *Investigative Ophthalmology & Visual Science*.

[9] Eppenberger L. S., Sturm V. (2020). The role of time exposed to outdoor light for myopia prevalence and progression: a literature review. *Clinical Ophthalmology*.

[10] Dirani M., Tong L., Gazzard G. (2009). Outdoor activity and myopia in Singapore teenage children. *British Journal of Ophthalmology*.

[11] Sun J. T., An M., Yan X. B., Li G. H., Wang D. B. (2018). Prevalence and related factors for myopia in school-aged children in qingdao. *Journal of Ophthalmology*.

[12] Jones-Jordan L. A., Sinnott L. T., Cotter S. A. (2012). Time outdoors, visual activity, and myopia progression in juvenile-onset myopes. *Investigative Ophthalmology & Visual Science*.

[13] Jacobsen N., Jensen H., Goldschmidt E. (2008). Does the level of physical activity in university students influence development and progression of myopia?—a 2-year prospective cohort study. *Investigative Ophthalmology & Visual Science*.

[14] Suhr Thykjaer A., Lundberg K., Grauslund J. (2017). Physical activity in relation to development and progression of myopia - a systematic review. *Acta Ophthalmologica*.

[15] Guggenheim J. A., Northstone K., McMahon G. (2012). Time outdoors and physical activity as predictors of incident myopia in childhood: a prospective cohort study. *Investigative Ophthalmology & Visual Science*.

[16] Saxena R., Vashist P., Tandon R. (2017). Incidence and progression of myopia and associated factors in urban school children in Delhi: the North India Myopia Study (NIM Study). *PLoS One*.

[17] Wu L. J., Wang Y. X., You Q. S. (2015). Risk factors of myopic shift among primary school children in beijing, China: a prospective study. *International Journal of Medical Sciences*.

[18] Hsu C. C., Huang N., Lin P. Y. (2017). Risk factors for myopia progression in second-grade primary school children in Taipei: a population-based cohort study. *British Journal of Ophthalmology*.

[19] Ma Y., Lin S., Zhu J. (2018). Different patterns of myopia prevalence and progression between internal migrant and local resident school children in Shanghai, China: a 2-year cohort study. *BMC Ophthalmology*.

[20] He X., Sankaridurg P., Xiong S. (2019). Shanghai time outside to reduce myopia trial: design and baseline data. *Clinical and Experimental Ophthalmology*.

[21] Schmid K. L., Wildsoet C. F. (1996). Effects on the compensatory responses to positive and negative lenses of intermittent lens wear and ciliary nerve section in chicks. *Vision Research*.

[22] Ashby R. (2016). Animal studies and the mechanism of myopia—protection by light?. *Optometry and Vision Science*.

[23] Morgan I. G., Ashby R. S. (2017). Bright light blocks the development of form deprivation myopia in mice, acting on D1 dopamine receptors. *Investigative Ophthalmology & Visual Science*.

[24] Huang H. M., Chang D. S. T., Wu P. C. (2015). The association between near work activities and myopia in children-A systematic review and meta-analysis. *PLoS One*.

[25] Enthoven C. A., Tideman J. W. L., Polling J. R., Yang-Huang J., Raat H., Klaver C. C. W. (2020). The impact of computer use on myopia development in childhood: the Generation R study. *Preventive Medicine*.

[26] Öner V., Bulut A., Oruç Y., Özgür G. (2016). Influence of indoor and outdoor activities on progression of myopia during puberty. *International Ophthalmology*.

[27] Pärssinen O., Lyyra A. L. (1993). Myopia and myopic progression among schoolchildren: a three-year follow-up study. *Investigative Ophthalmology & Visual Science*.

[28] Tan N. W. H., Saw S. M., Lam D. S. C., Cheng H. M., Rajan U., Chew S. J. (2000). Temporal variations in myopia progression in Singaporean children within an academic year. *Optometry and Vision Science*.

[29] Saw S. M., Nieto F. J., Katz J., Schein O. D., Levy B., Chew S. J. (2000). Factors related to the progression of myopia in Singaporean children. *Optometry and Vision Science*.

[30] Ip J. M., Saw S. M., Rose K. A. (2008). Role of near work in myopia: findings in a sample of Australian school children. *Investigative Ophthalmology & Visual Science*.

[31] Wong C. W., Tsai A., Jonas J. B. (2021). Digital screen time during the COVID-19 pandemic: risk for a further myopia boom?. *American Journal of Ophthalmology*.

[32] Lanca C., Saw S. M. (2020). The association between digital screen time and myopia: a systematic review. *Ophthalmic and Physiological Optics*.

[33] Jiang Y., Zhu Z., Tan X. (2022). Effect of repeated low-level red-light therapy for myopia control in children: a multicenter randomized controlled trial. *Ophthalmology*.

[34] Schaeffel F., Troilo D., Wallman J., Howland H. C. (1990). Developing eyes that lack accommodation grow to compensate for imposed defocus. *Visual Neuroscience*.

[35] Wildsoet C. F. (2003). Neural pathways subserving negative lens-induced emmetropization in chicks--insights from selective lesions of the optic nerve and ciliary nerve. *Current Eye Research*.

[36] Qu J., Zhou X., Xie R. (2006). The presence of m1 to m5 receptors in human sclera: evidence of the sclera as a potential site of action for muscarinic receptor antagonists. *Current Eye Research*.

[37] Barathi V. A., Weon S. R., Beuerman R. W. (2009). Expression of muscarinic receptors in human and mouse sclera and their role in the regulation of scleral fibroblasts proliferation. *Molecular Vision*.

[38] Carr B. J., Stell W. K. (2016). Nitric oxide (NO) mediates the inhibition of form-deprivation myopia by atropine in chicks. *Scientific Reports*.

[39] Barathi V. A., Chaurasia S. S., Poidinger M. (2014). Involvement of GABA transporters in atropine-treated myopic retina as revealed by iTRAQ quantitative proteomics. *Journal of Proteome Research*.

[40] Carr B. J., Mihara K., Ramachandran R. (2018). Myopia-inhibiting concentrations of muscarinic receptor antagonists block Activation of Alpha2A-adrenoceptors in vitro. *Investigative Ophthalmology & Visual Science*.

[41] Prepas S. B. (2008). Light, literacy and the absence of ultraviolet radiation in the development of myopia. *Medical Hypotheses*.

[42] Shih Y. F., Chen C. H., Chou A. C., Ho T. C., Lin L. L. K., Hung P. T. (1999). Effects of different concentrations of atropine on controlling myopia in myopic children. *Journal of Ocular Pharmacology and Therapeutics*.

[43] Chua W. H., Balakrishnan V., Chan Y. H. (2006). Atropine for the treatment of childhood myopia. *Ophthalmology*.

[44] Yi S., Huang Y., Yu S. Z., Chen X. J., Yi H., Zeng X. L. (2015). Therapeutic effect of atropine 1% in children with low myopia. *Journal of American Association for Pediatric Ophthalmology and Strabismus*.

[45] Wang Y. R., Bian H. L., Wang Q. (2017). Atropine 0.5% eyedrops for the treatment of children with low myopia: a randomized controlled trial. *Medicine (Baltimore)*.

[46] Tong L., Huang X. L., Koh A. L. T., Zhang X., Tan D. T. H., Chua W. H. (2009). Atropine for the treatment of childhood myopia: effect on myopia progression after cessation of atropine. *Ophthalmology*.

[47] Chia A., Chua W. H., Cheung Y. B. (2012). Atropine for the treatment of childhood myopia: safety and efficacy of 0.5%, 0.1%, and 0.01% doses (atropine for the treatment of myopia 2). *Ophthalmology*.

[48] Chia A., Chua W. H., Wen L., Fong A., Goon Y. Y., Tan D. (2014). Atropine for the treatment of childhood myopia: changes after stopping atropine 0.01%, 0.1% and 0.5%. *American Journal of Ophthalmology*.

[49] Yam J. C., Jiang Y., Tang S. M. (2019). Low-concentration atropine for myopia progression (LAMP) study: a randomized, double-blinded, placebo-controlled trial of 0.05%, 0.025%, and 0.01% atropine eye drops in myopia control. *Ophthalmology*.

[50] Yam J. C., Li F. F., Zhang X. (2020). Two-year clinical trial of the low-concentration atropine for myopia progression (LAMP) study: phase 2 report. *Ophthalmology*.

[51] Yam J. C., Zhang X. J., Zhang Y. (2022). Three-year clinical trial of low-concentration atropine for myopia progression (LAMP) study: continued versus washout. *Ophthalmology*.

[52] Siatkowski R. M., Cotter S., Miller J. M. (2004). Safety and efficacy of 2% pirenzepine ophthalmic gel in children with myopia: a 1-year, multicenter, double-masked, placebo-controlled parallel study. *Archives of Ophthalmology*.

[53] Siatkowski R. M., Cotter S. A., Crockett R. S., Miller J. M., Novack G. D., Zadnik K. (2008). Two-year multicenter, randomized, double-masked, placebo-controlled, parallel safety and efficacy study of 2% pirenzepine ophthalmic gel in children with myopia. *Journal of American Association for Pediatric Ophthalmology and Strabismus*.

[54] Tan D. T. H., Lam D. S., Chua W. H., Shu-Ping D. F., Crockett R. S. (2005). One-year multicenter, double-masked, placebo-controlled, parallel safety and efficacy study of 2% pirenzepine ophthalmic gel in children with myopia. *Ophthalmology*.

[55] Trier K., Olsen E. B., Kobayashi T., Ribel-Madsen S. M. (1999). Biochemical and ultrastructural changes in rabbit sclera after treatment with 7-methylxanthine, theobromine, acetazolamide, or L-ornithine. *British Journal of Ophthalmology*.

[56] Trier K., Munk Ribel-Madsen S., Cui D., Brøgger Christensen S. (2008). Systemic 7-methylxanthine in retarding axial eye growth and myopia progression: a 36-month pilot study. *Journal of Ocular Biology, Diseases, and Informatics*.

[57] Nie H. H., Huo L. J., Yang X. (2012). Effects of 7-methylxanthine on form-deprivation myopia in pigmented rabbits. *International Journal of Ophthalmology*.

[58] Cui D., Trier K., Zeng J. (2011). Effects of 7-methylxanthine on the sclera in form deprivation myopia in Guinea pigs. *Acta Ophthalmologica*.

[59] Singh D. (1995). Photorefractive keratectomy in pediatric patients. *Journal of Cataract & Refractive Surgery*.

[60] Stahl E. D. (2014). Pediatric refractive surgery. *Pediatric Clinics of North America*.

[61] Høeg T. B., Moldow B., Ellervik C. (2015). Danish rural eye study: the association of preschool vision screening with the prevalence of amblyopia. *Acta Ophthalmologica*.

[62] Weakley D. R. (2001). The association between nonstrabismic anisometropia, amblyopia, and subnormal binocularity. *Ophthalmology*.

[63] Cobb C. J., Russell K., Cox A., MacEwen C. J., Cox A. (2002). Factors influencing visual outcome in anisometropic amblyopes. *British Journal of Ophthalmology*.

[64] Daoud Y. J., Hutchinson A., Wallace D. K., Song J., Kim T. (2009). Refractive surgery in children: treatment options, outcomes, and controversies. *American Journal of Ophthalmology*.

[65] Paysse E. A. (2004). Photorefractive keratectomy for anisometropic amblyopia in children. *Transactions of the American Ophthalmological Society*.

[66] Tychsen L. (2008). Refractive surgery for children: excimer laser, phakic intraocular lens, and clear lens extraction. *Current Opinion in Ophthalmology*.

[67] Pediatric Eye Disease Investigator Group (2002). A randomized trial of atropine vs. patching for treatment of moderate amblyopia in children. *Archives of Ophthalmology*.

[68] Holmes J. M., Kraker R. T., Beck R. W. (2003). A randomized trial of prescribed patching regimens for treatment of severe amblyopia in children. *Ophthalmology*.

[69] Nano H. D., Muzzin S., Irigaray F. L. (1997). Excimer laser photorefractive keratectomy in pediatric patients. *Journal of Cataract & Refractive Surgery*.

[70] Rashad K. M. (1999). Laser in situ keratomileusis for myopic anisometropia in children. *Journal of Refractive Surgery*.

[71] Alió J. L., Artola A., Claramonte P., Ayala M. J., Chipont E. (1998). Photorefractive keratectomy for pediatric myopic anisometropia. *Journal of Cataract & Refractive Surgery*.

[72] Agarwal A., Agarwal A., Agarwal T., Azim Siraj A., Narang P., Narang S. (2000). Results of pediatric laser in situ keratomileusis. *Journal of Cataract & Refractive Surgery*.

[73] Nucci P., Drack A. V. (2001). Refractive surgery for unilateral high myopia in children. *Journal of American Association for Pediatric Ophthalmology and Strabismus*.

[74] Nassaralla B. R. A., Nassaralla J. J. (2001). Laser in situ keratomileusis in children 8 to 15 years old. *Journal of Refractive Surgery*.

[75] Astle W. F., Huang P. T., Ells A. L., Cox R. G., Deschenes M. C., Vibert H. M. (2002). Photorefractive keratectomy in children. *Journal of Cataract & Refractive Surgery*.

[76] Astle W. F., Huang P. T., Ingram A. D., Farran P. R. (2004). Laser-assisted subepithelial keratectomy in children. *Journal of Cataract & Refractive Surgery*.

[77] Autrata R., Rehurek J. (2004). Laser-assisted subepithelial keratectomy and photorefractive keratectomy versus conventional treatment of myopic anisometropic amblyopia in children. *Journal of Cataract & Refractive Surgery*.

[78] O’Keefe M. (2004). LASIK surgery in children. *British Journal of Ophthalmology*.

[79] Tychsen L., Packwood E., Berdy G. (2005). Correction of large amblyopiogenic refractive errors in children using the excimer laser. *Journal of American Association for Pediatric Ophthalmology and Strabismus*.

[80] Paysse E. A., Coats D. K., Hussein M. A. W., Hamill M. B., Koch D. D. (2006). Long-term outcomes of photorefractive keratectomy for anisometropic amblyopia in children. *Ophthalmology*.

[81] Astle W. F., Rahmat J., Ingram A. D., Huang P. T. (2007). Laser-assisted subepithelial keratectomy for anisometropic amblyopia in children: outcomes at 1 year. *Journal of Cataract & Refractive Surgery*.

[82] Yin Z. Q., Wang H., Yu T., Ren Q., Chen L. (2007). Facilitation of amblyopia management by laser in situ keratomileusis in high anisometropic hyperopic and myopic children. *Journal of American Association for Pediatric Ophthalmology and Strabismus*.

[83] Autrata R., Rehurek J. (2003). Clinical results of excimer laser photorefractive keratectomy for high myopic anisometropia in children: four-year follow-up. *Journal of Cataract & Refractive Surgery*.

[84] Phillips C. B., Prager T. C., McClellan G., Mintz-Hittner H. A. (2004). Laser in situ keratomileusis for treated anisometropic amblyopia in awake, autofixating pediatric and adolescent patients. *Journal of Cataract & Refractive Surgery*.

[85] Magli A., Iovine A., Gagliardi V., Fimiani F., Nucci P. (2008). Photorefractive keratectomy for myopic anisometropia: a retrospective study on 18 children. *European Journal of Ophthalmology*.

[86] Tychsen L., Hoekel J. (2006). Refractive surgery for high bilateral myopia in children with neurobehavioral disorders: 2. Laser-assisted subepithelial keratectomy (LASEK). *Journal of American Association for Pediatric Ophthalmology and Strabismus*.

[87] Alió J. L., Wolter N. V., Piñero D. P. (2011). Pediatric refractive surgery and its role in the treatment of amblyopia: meta-analysis of the peer-reviewed literature. *Journal of Refractive Surgery*.

[88] Paysse E. A., Tychsen L., Stahl E. (2012). Pediatric refractive surgery: corneal and intraocular techniques and beyond. *Journal of American Association for Pediatric Ophthalmology and Strabismus*.

[89] Mahfouz A. K. M., Khalaf M. A. (2005). Comparative study of 2 anesthesia techniques for pediatric refractive surgery. *Journal of Cataract & Refractive Surgery*.

[90] Paysse E. A., Hussein M. A. W., Koch D. D. (2003). Successful implementation of a protocol for photorefractive keratectomy in children requiring anesthesia. *Journal of Cataract & Refractive Surgery*.

[91] Brown S. M. (2009). Pediatric refractive surgery. *Archives of Ophthalmology*.

[92] Stahl E. D. (2017). Pediatric refractive surgery. *Current Opinion in Ophthalmology*.

[93] Knox Cartwright N. E., Tyrer J. R., Marshall J. (2011). Age-related differences in the elasticity of the human cornea. *Investigative Ophthalmology & Visual Science*.

[94] Lesueur L. C., Arne J. L. (1999). Phakic posterior chamber lens implantation in children with high myopia 1. *Journal of Cataract & Refractive Surgery*.

[95] Moran S., O’Keefe M. (2013). The role of phakic intraocular lens implants in treatment of high-refractive errors and amblyopia in children. *Ophthalmology and Therapy*.

[96] Pirouzian A. (2010). Pediatric phakic intraocular lens surgery: review of clinical studies. *Current Opinion in Ophthalmology*.

[97] Pirouzian A., Ip K. C., O’Halloran H. S. (2009). Phakic anterior chamber intraocular lens (Verisyse) implantation in children for treatment of severe ansiometropia myopia and amblyopia: six-month pilot clincial trial and review of literature. *Clinical Ophthalmology*.

[98] Ryan A., Hartnett C., Lanigan B., O’Keefe M. (2012). Foldable iris-fixated intraocular lens implantation in children. *Acta Ophthalmologica*.

[99] Alió J. L., Toffaha B. T., Laria C., Piñero D. P. (2011). Phakic intraocular lens implantation for treatment of anisometropia and amblyopia in children: 5-year follow-up. *Journal of Refractive Surgery*.

[100] Pirouzian A., Ip K. C. (2010). Anterior chamber phakic intraocular lens implantation in children to treat severe anisometropic myopia and amblyopia: 3-year clinical results. *Journal of Cataract & Refractive Surgery*.

[101] Emara K. E., Al Abdulsalam O., Al Habash A. (2015). Implantation of spherical and toric copolymer phackic intraocular lens to manage amblyopia due to anisometropic hyperopia and myopia in pediatric patients. *Journal of Cataract & Refractive Surgery*.

[102] Zhang J., Li J. R., Chen Z. D., Yu M. B., Yu K. M. (2016). Phakic posterior chamber intraocular lens for unilateral high myopic amblyopia in Chinese pediatric patients. *International Journal of Ophthalmology*.

[103] Tychsen L., Faron N., Hoekel J. (2017). Phakic intraocular collamer lens (visian ICL) implantation for correction of myopia in spectacle-aversive special needs children. *American Journal of Ophthalmology*.

[104] Hu P. H., Guo X. M. (2012). Effects of different degree correction of refractive error in young myopia patients. *Guoji Yanke Zazhi*.

[105] Sun Y. Y., Li S. M., Li S. Y. (2017). Effect of uncorrection versus full correction on myopia progression in 12-year-old children. *Graefes Archive for Clinical and Experimental Ophthalmology*.

[106] Ong E., Grice K., Held R., Thorn F., Gwiazda J. (1999). Effects of spectacle intervention on the progression of myopia in children. *Optometry and Vision Science*.

[107] Adler D., Millodot M. (2006). The possible effect of undercorrection on myopic progression in children. *Clinical and Experimental Optometry*.

[108] Shaikh A. W., Siegwart J. T., Norton T. T. (1999). Effect of interrupted lens wear on compensation for a minus lens in tree shrews. *Optometry and Vision Science*.

[109] Smith III E. L., Hung L. F. (1999). The role of optical defocus in regulating refractive development in infant monkeys. *Vision Research*.

[110] Tokoro T., Kabe S. (1965). Treatment of the myopia and the changes in optical components. Report II. Full-or under-correction of myopia by glasses. *Nippon Ganka Gakkai Zasshi*.

[111] Li S. Y., Li S. M., Zhou Y. H. (2015). Effect of undercorrection on myopia progression in 12-year-old children. *Graefes Archive for Clinical and Experimental Ophthalmology*.

[112] Koomson N. Y., Amedo A. O., Opoku-Baah C., Ampeh P. B., Ankamah E., Bonsu K. (2016). Relationship between reduced accommodative lag and myopia progression. *Optometry and Vision Science*.

[113] Chung K., Mohidin N., O’Leary D. J. (2002). Undercorrection of myopia enhances rather than inhibits myopia progression. *Vision Research*.

[114] Chen Y. H. (2014). Clinical observation of the development of juvenile myopia wearing glasses with full correction and under-correction. *Guoji Yanke Zazhi*.

[115] Vasudevan B., Esposito C., Peterson C., Coronado C., Ciuffreda K. J. (2014). Under-correction of human myopia--is it myopigenic?: a retrospective analysis of clinical refraction data. *Journal of Optometry*.

[116] Phillips J. R. (2005). Monovision slows juvenile myopia progression unilaterally. *British Journal of Ophthalmology*.

[117] Goss D. A. (1984). Overcorrection as a means of slowing myopic progression. *Optometry and Vision Science*.

[118] Wallman J., Winawer J. (2004). Homeostasis of eye growth and the question of myopia. *Neuron*.

[119] Goss D. A., Rainey B. B. (1999). Relationship of accommodative response and nearpoint phoria in a sample of myopic children. *Optometry and Vision Science*.

[120] Gwiazda J., Thorn F., Bauer J., Held R. (1993). Myopic children show insufficient accommodative response to blur. *Investigative Ophthalmology & Visual Science*.

[121] Mutti D. O., Zadnik K., Fusaro R. E., Friedman N. E., Sholtz R. I., Adams A. J. (1998). Optical and structural development of the crystalline lens in childhood. *Investigative Ophthalmology & Visual Science*.

[122] Zadnik K., Mutti D. O., Fusaro R. E., Adams A. J. (1995). Longitudinal evidence of crystalline lens thinning in children. *Investigative Ophthalmology & Visual Science*.

[123] Mutti D. O., Mitchell G. L., Hayes J. R. (2006). Accommodative lag before and after the onset of myopia. *Investigative Ophthalmology & Visual Science*.

[124] Mutti D. O., Hayes J. R., Mitchell G. L. (2007). Refractive error, axial length, and relative peripheral refractive error before and after the onset of myopia. *Investigative Ophthalmology & Visual Science*.

[125] Berntsen D. A., Mutti D. O., Zadnik K. (2010). Study of theories about myopia progression (STAMP) design and baseline data. *Optometry and Vision Science*.

[126] Gwiazda J., Bauer J., Thorn F., Held R. (1995). A dynamic relationship between myopia and blur-driven accommodation in school-aged children. *Vision Research*.

[127] Gwiazda J., Thorn F., Held R. (2005). Accommodation, accommodative convergence, and response AC/A ratios before and at the onset of myopia in children. *Optometry and Vision Science*.

[128] Gwiazda J. E., Hyman L., Norton T. T. (2004). Accommodation and related risk factors associated with myopia progression and their interaction with treatment in COMET children. *Investigative Ophthalmology & Visual Science*.

[129] Gwiazda J., Hyman L., Hussein M. (2003). A randomized clinical trial of progressive addition lenses versus single vision lenses on the progression of myopia in children. *Investigative Ophthalmology & Visual Science*.

[130] Correction of Myopia Evaluation Trial 2 Study Group for the Pediatric Eye Disease Investigator Group (2011). Progressive-addition lenses versus single-vision lenses for slowing progression of myopia in children with high accommodative lag and near esophoria. *Investigative Ophthalmology & Visual Science*.

[131] Cheng D., Woo G. C., Drobe B., Schmid K. L. (2014). Effect of bifocal and prismatic bifocal spectacles on myopia progression in children: three-year results of a randomized clinical trial. *JAMA Ophthalmology*.

[132] Smith E. L., Kee C. S., Ramamirtham R., Qiao-Grider Y., Hung L. F. (2005). Peripheral vision can influence eye growth and refractive development in infant monkeys. *Investigative Ophthalmology & Visual Science*.

[133] Smith E. L., Ramamirtham R., Qiao-Grider Y. (2007). Effects of foveal ablation on emmetropization and form-deprivation myopia. *Investigative Ophthalmology & Visual Science*.

[134] Smith E. L., Hung L. F., Huang J. (2009). Relative peripheral hyperopic defocus alters central refractive development in infant monkeys. *Vision Research*.

[135] Benavente-Pérez A., Nour A., Troilo D. (2014). Axial eye growth and refractive error development can be modified by exposing the peripheral retina to relative myopic or hyperopic defocus. *Investigative Ophthalmology & Visual Science*.

[136] Liu Y., Wildsoet C. (2011). The effect of two-zone concentric bifocal spectacle lenses on refractive error development and eye growth in young chicks. *Investigative Ophthalmology & Visual Science*.

[137] Liu Y., Wildsoet C. (2012). The effective add inherent in 2-zone negative lenses inhibits eye growth in myopic young chicks. *Investigative Ophthalmology & Visual Science*.

[138] Tepelus T. C., Vazquez D., Seidemann A., Uttenweiler D., Schaeffel F. (2012). Effects of lenses with different power profiles on eye shape in chickens. *Vision Research*.

[139] Neil Charman W., Radhakrishnan H. (2010). Peripheral refraction and the development of refractive error: a review. *Ophthalmic and Physiological Optics*.

[140] Berntsen D. A., Barr C. D., Mutti D. O., Zadnik K. (2013). Peripheral defocus and myopia progression in myopic children randomly assigned to wear single vision and progressive addition lenses. *Investigative Ophthalmology & Visual Science*.

[141] Lam C. S. Y., Tang W. C., Tse D. Y. Y. (2020). Defocus incorporated multiple segments (DIMS) spectacle lenses slow myopia progression: a 2-year randomised clinical trial. *British Journal of Ophthalmology*.

[142] Lam C. S., Tang W. C., Lee P. H. (2021). Myopia control effect of defocus incorporated multiple segments (DIMS) spectacle lens in Chinese children: results of a 3-year follow-up study. *British Journal of Ophthalmology*.

[143] Zhang H., Lam C. S. Y., Tang W. C. (2022). Myopia control effect is influenced by baseline relative peripheral refraction in children wearing defocus incorporated multiple segments (DIMS) spectacle lenses. *Journal of Clinical Medicine*.

[144] Tse D. Y., Lam C. S., Guggenheim J. A. (2007). Simultaneous defocus integration during refractive development. *Investigative Ophthalmology & Visual Science*.

[145] Stone R. A., Flitcroft D. I. (2004). Ocular shape and myopia. *Annals Academy of Medicine Singapore*.

[146] Zhang H. Y., Lam C. S. Y., Tang W. C., Leung M., To C. H. (2020). Defocus incorporated multiple segments spectacle lenses changed the relative peripheral refraction: a 2-year randomized clinical trial. *Investigative Ophthalmology & Visual Science*.

[147] Sankaridurg P., Holden B., Smith E. (2011). Decrease in rate of myopia progression with a contact lens designed to reduce relative peripheral hyperopia: one-year results. *Investigative Ophthalmology & Visual Science*.

[148] Li Y., Fu Y., Wang K., Liu Z., Shi X., Zhao M. (2020). Evaluating the myopia progression control efficacy of defocus incorporated multiple segments (DIMS) lenses and apollo progressive addition spectacle lenses (PALs) in 6- to 12-year-old children: study protocol for a prospective, multicenter, randomized controlled trial. *Trials*.

[149] Kang P. (2018). Optical and pharmacological strategies of myopia control. *Clinical and Experimental Optometry*.

[150] Sankaridurg P. (2017). Contact lenses to slow progression of myopia. *Clinical and Experimental Optometry*.

[151] Schaeffel F., Glasser A., Howland H. C. (1988). Accommodation, refractive error and eye growth in chickens. *Vision Research*.

[152] Norton T. T., Siegwart J. T. (1995). Animal models of emmetropization: matching axial length to the focal plane. *Journal of the American Optometric Association*.

[153] Troilo D., Quinn N., Baker K. (2007). Accommodation and induced myopia in marmosets. *Vision Research*.

[154] Howlett M. H. C., McFadden S. A. (2009). Spectacle lens compensation in the pigmented Guinea pig. *Vision Research*.

[155] Graham B., Judge S. J. (1999). The effects of spectacle wear in infancy on eye growth and refractive error in the marmoset (*Callithrix jacchus*). *Vision Research*.

[156] Hung L. F., Crawford M. L., Smith E. L. (1995). Spectacle lenses alter eye growth and the refractive status of young monkeys. *Nature Medicine*.

[157] Kelly T. S., Chatfield C., Tustin G. (1975). Clinical assessment of the arrest of myopia. *British Journal of Ophthalmology*.

[158] Perrigin J., Perrigin D., Quintero S., Grosvenor T. (1990). Silicone-acrylate contact lenses for myopia control: 3-year results. *Optometry and Vision Science*.

[159] Walline J. J., Mutti D. O., Jones L. A. (2001). The contact lens and myopia progression (CLAMP) study: design and baseline data. *Optometry and Vision Science*.

[160] Katz J., Schein O. D., Levy B. (2003). A randomized trial of rigid gas permeable contact lenses to reduce progression of children’s myopia. *American Journal of Ophthalmology*.

[161] Atchison D. A. (2006). Optical models for human myopic eyes. *Vision Research*.

[162] Walline J. J., Jones L. A., Sinnott L. (2008). A randomized trial of the effect of soft contact lenses on myopia progression in children. *Investigative Ophthalmology & Visual Science*.

[163] Horner D. G., Soni P. S., Salmon T. O., Swartz T. S. (1999). Myopia progression in adolescent wearers of soft contact lenses and spectacles. *Optometry and Vision Science*.

[164] Ruiz-Pomeda A., Pérez-Sánchez B., Valls I., Prieto-Garrido F. L., Gutiérrez-Ortega R., Villa-Collar C. (2018). MiSight assessment study spain (MASS). A 2-year randomized clinical trial. *Graefes Archive for Clinical and Experimental Ophthalmology*.

[165] Sankaridurg P., Bakaraju R. C., Naduvilath T. (2019). Myopia control with novel central and peripheral plus contact lenses and extended depth of focus contact lenses: 2 year results from a randomised clinical trial. *Ophthalmic and Physiological Optics*.

[166] Li S. M., Kang M. T., Wu S. S. (2017). Studies using concentric ring bifocal and peripheral add multifocal contact lenses to slow myopia progression in school-aged children: a meta-analysis. *Ophthalmic and Physiological Optics*.

[167] Sun Y., Xu F., Zhang T. (2015). Orthokeratology to control myopia progression: a meta-analysis. *PLoS One*.

[168] Huang J., Wen D., Wang Q. (2016). Efficacy comparison of 16 interventions for myopia control in children: a network meta-analysis. *Ophthalmology*.

[169] Anstice N. S., Phillips J. R. (2011). Effect of dual-focus soft contact lens wear on axial myopia progression in children. *Ophthalmology*.

[170] Hasebe S., Ohtsuki H., Nonaka T. (2008). Effect of progressive addition lenses on myopia progression in Japanese children: a prospective, randomized, double-masked, crossover trial. *Investigative Ophthalmology & Visual Science*.

[171] Berntsen D. A., Sinnott L. T., Mutti D. O., Zadnik K. (2012). A randomized trial using progressive addition lenses to evaluate theories of myopia progression in children with a high lag of accommodation. *Investigative Ophthalmology & Visual Science*.

[172] Fulk G. W., Cyert L. A., Parker D. E. (2000). A randomized trial of the effect of single-vision vs. bifocal lenses on myopia progression in children with esophoria. *Optometry and Vision Science*.

[173] Aller T. A., Liu M., Wildsoet C. F. (2016). Myopia control with bifocal contact lenses: a randomized clinical trial. *Optometry and Vision Science*.

[174] Cheng X., Xu J., Chehab K., Exford J., Brennan N. (2016). Soft contact lenses with positive spherical aberration for myopia control. *Optometry and Vision Science*.

[175] Fujikado T., Ninomiya S., Kobayashi T., Suzaki A., Nakada M., Nishida K. (2014). Effect of low-addition soft contact lenses with decentered optical design on myopia progression in children: a pilot study. *Clinical Ophthalmology*.

[176] Lam C. S. Y., Tang W. C., Tse D. Y. Y., Tang Y. Y., To C. H. (2014). Defocus incorporated soft contact (DISC) lens slows myopia progression in Hong Kong Chinese schoolchildren: a 2-year randomised clinical trial. *British Journal of Ophthalmology*.

[177] Pauné J., Morales H., Armengol J., Quevedo L., Faria-Ribeiro M., González-Méijome J. M. (2015). Myopia control with a novel peripheral gradient soft lens and orthokeratology: a 2-year clinical trial. *BioMed Research International*.

[178] Walline J. J., Greiner K. L., McVey M. E., Jones-Jordan L. A. (2013). Multifocal contact lens myopia control. *Optometry and Vision Science*.

[179] Walline J. J., Walker M. K., Mutti D. O. (2020). Effect of high add power, medium add power, or single-vision contact lenses on myopia progression in children: the BLINK randomized clinical trial. *JAMA*.

[180] Kerns R. L. (1976). Research in orthokeratology. Part I: introduction and background. *Journal of the American Optometric Association*.

[181] Wesley N., Jessen G. (1960). *Advanced Techniques in Contact Lens Fitting*.

[182] Swarbrick H. A., Wong G., O’Leary D. J. (1998). Corneal response to orthokeratology. *Optometry and Vision Science*.

[183] Tsukiyama J., Miyamoto Y., Higaki S., Fukuda M., Shimomura Y. (2008). Changes in the anterior and posterior radii of the corneal curvature and anterior chamber depth by orthokeratology. *Eye and Contact Lens: Science and Clinical Practice*.

[184] Chen R., Mao X., Jiang J. (2017). The relationship between corneal biomechanics and anterior segment parameters in the early stage of orthokeratology: a pilot study. *Medicine (Baltimore)*.

[185] Sankaridurg P., Conrad F., Tran H., Zhu J. (2018). Controlling progression of myopia: optical and pharmaceutical strategies. *Asia-Pacific Journal of Ophthalmology (Philadelphia, Pa.)*.

[186] Cheung S. W., Boost M. V., Cho P. (2019). Pre-treatment observation of axial elongation for evidence-based selection of children in Hong Kong for myopia control. *Contact Lens and Anterior Eye*.

[187] Wen D., Huang J., Chen H. (2015). Efficacy and acceptability of orthokeratology for slowing myopic progression in children: a systematic review and meta-analysis. *Journal of Ophthalmology*.

[188] Si J. K., Tang K., Bi H. S., Guo D. D., Guo J. G., Wang X. R. (2015). Orthokeratology for myopia control: a meta-analysis. *Optometry and Vision Science*.

[189] Koffler B. H., Sears J. J. (2013). Myopia control in children through refractive therapy gas permeable contact lenses: is it for real?. *American Journal of Ophthalmology*.

[190] https://www.clspectrum.com/issues/2017/january/international-contact-lens-prescribing-in-2016.

[191] Cho P., Cheung S. W., Edwards M. (2005). The longitudinal orthokeratology research in children (LORIC) in Hong Kong: a pilot study on refractive changes and myopic control. *Current Eye Research*.

[192] Walline J. J., Jones L. A., Sinnott L. T. (2009). Corneal reshaping and myopia progression. *British Journal of Ophthalmology*.

[193] Santodomingo-Rubido J., Villa-Collar C., Gilmartin B., Gutiérrez-Ortega R. (2012). Myopia control with orthokeratology contact lenses in Spain: refractive and biometric changes. *Investigative Ophthalmology & Visual Science*.

[194] Charm J., Cho P. (2013). High myopia-partial reduction ortho-k: a 2-year randomized study. *Optometry and Vision Science*.

[195] Cho P., Cheung S. W. (2012). Retardation of myopia in Orthokeratology (ROMIO) study: a 2-year randomized clinical trial. *Investigative Ophthalmology & Visual Science*.

[196] Charm J., Cho P. (2013). High myopia-partial reduction orthokeratology (HM-PRO): study design. *Contact Lens and Anterior Eye*.

[197] Swarbrick H. A., Alharbi A., Watt K., Lum E., Kang P. (2015). Myopia control during orthokeratology lens wear in children using a novel study design. *Ophthalmology*.

[198] Na M., Yoo A. (2018). The effect of orthokeratology on axial length elongation in children with myopia: contralateral comparison study. *Japanese Journal of Ophthalmology*.

[199] Zhang Y., Chen Y. G. (2018). Comparison of myopia control between toric and spherical periphery design orthokeratology in myopic children with moderate-to-high corneal astigmatism. *International Journal of Ophthalmology*.

[200] Wan L., Wei C. C., Chen C. S. (2018). The synergistic effects of orthokeratology and atropine in slowing the progression of myopia. *Journal of Clinical Medicine*.

[201] Kinoshita N., Konno Y., Hamada N., Kanda Y., Shimmura-Tomita M., Kakehashi A. (2018). Additive effects of orthokeratology and atropine 0.01% ophthalmic solution in slowing axial elongation in children with myopia: first year results. *Japanese Journal of Ophthalmology*.

[202] Walline J. J., Lindsley K., Vedula S. S., Cotter S. A., Mutti D. O., Twelker J. D. (2011). Interventions to slow progression of myopia in children. *Cochrane Database of Systematic Reviews*.

[203] Kakita T., Hiraoka T., Oshika T. (2011). Influence of overnight orthokeratology on axial elongation in childhood myopia. *Investigative Ophthalmology & Visual Science*.

[204] Wang B., Naidu R. K., Qu X. (2017). Factors related to axial length elongation and myopia progression in orthokeratology practice. *PLoS One*.

[205] Zhong Y., Chen Z., Xue F., Zhou J., Niu L., Zhou X. (2014). Corneal power change is predictive of myopia progression in orthokeratology. *Optometry and Vision Science*.

[206] Chen Z., Niu L., Xue F. (2012). Impact of pupil diameter on axial growth in orthokeratology. *Optometry and Vision Science*.

[207] Lee Y. C., Wang J. H., Chiu C. J. (2017). Effect of Orthokeratology on myopia progression: twelve-year results of a retrospective cohort study. *BMC Ophthalmology*.

[208] Walline J. J., Jones L. A., Rah M. J. (2007). Contact lenses in pediatrics (CLIP) study: chair time and ocular health. *Optometry and Vision Science*.

[209] Walline J. J., Long S., Zadnik K. (2004). Daily disposable contact lens wear in myopic children. *Optometry and Vision Science*.

